# *In Vivo* and *In Vitro* Detection of Luminescent and Fluorescent *Lactobacillus reuteri* and Application of Red Fluorescent mCherry for Assessing Plasmid Persistence

**DOI:** 10.1371/journal.pone.0151969

**Published:** 2016-03-22

**Authors:** Shokoufeh Karimi, David Ahl, Evelina Vågesjö, Lena Holm, Mia Phillipson, Hans Jonsson, Stefan Roos

**Affiliations:** 1 Department of Microbiology, Uppsala BioCenter, Swedish University of Agricultural Sciences, Uppsala, Sweden; 2 Department of Medical Cell Biology, Biomedical Center, Uppsala University, Uppsala, Sweden; Commissariat a l'Energie Atomique(cea), FRANCE

## Abstract

*Lactobacillus reuteri* is a symbiont that inhabits the gastrointestinal (GI) tract of mammals, and several strains are used as probiotics. After introduction of probiotic strains in a complex ecosystem like the GI tract, keeping track of them is a challenge. The main objectives of this study were to introduce reporter proteins that would enable *in vivo* and *in vitro* detection of *L*. *reuteri* and increase knowledge about its interactions with the host. We describe for the first time cloning of codon-optimized reporter genes encoding click beetle red luciferase (CBRluc) and red fluorescent protein mCherry in *L*. *reuteri* strains ATCC PTA 6475 and R2LC. The plasmid persistence of mCherry-expressing lactobacilli was evaluated by both flow cytometry (FCM) and conventional plate count (PC), and the plasmid loss rates measured by FCM were lower overall than those determined by PC. Neutralization of pH and longer induction duration significantly improved the mCherry signal. The persistency, dose-dependent signal intensity and localization of the recombinant bacteria in the GI tract of mice were studied with an *in vivo* imaging system (IVIS), which allowed us to detect fluorescence from 6475-CBRluc-mCherry given at a dose of 1×10^10^ CFU and luminescence signals at doses ranging from 1×10^5^ to 1×10^10^ CFU. Both 6475-CBRluc-mCherry and R2LC-CBRluc were localized in the colon 1 and 2 h after ingestion, but the majority of the latter were still found in the stomach, possibly reflecting niche specificity for R2LC. Finally, an *in vitro* experiment showed that mCherry-producing R2LC adhered efficiently to the intra cellular junctions of cultured IPEC-J2 cells. In conclusion, the two reporter genes CBRluc and mCherry were shown to be suitable markers for biophotonic imaging (BPI) of *L*. *reuteri* and may provide useful tools for future studies of *in vivo* and *in vitro* interactions between the bacteria and the host.

## Introduction

*Lactobacillus reuteri* is a lactic acid bacterium with an unusually broad host range and the species is considered a ubiquitous indigenous inhabitant of the gastrointestinal (GI) tract of birds and mammals [1], including humans [2, 3]. The bacterium has also been found in naturally discharged fluid of other organs such as the female utero-vaginal tract [4] and mammary ducts (milk) [4, 5]. As for many other members of the *Lactobacillus* genus, research interest in *L*. *reuteri* has grown due to its potential to improve health, and strains of the species have been marketed as probiotics. Some strains of *L*. *reuteri* have shown good health-promoting efficacy in both animal models [6, 7] and human clinical trials [8, 9]. *Lactobacillus reuteri* ATCC PTA 6475 is one of the most widely investigated strains and several animal studies have demonstrated its health benefits, including anti-inflammatory effect [10, 11], integumentary health promoting effect on aged mice including improvement of fur and skin quality [12], increased bone mass as a result of reduction of osteoclastogenesis [13] and osteoblastic activity [14].

A human trial has also demonstrated an inhibitory effect of a combination of *L*. *reuteri* ATCC PTA 6475 and *L*. *reuteri* DSM 17938 on *Helicobacter pylori* growth and, when administered with eradication therapy, reduced antibiotic-associated side-effects [15]. R2LC is a strain of *L*. *reuteri* isolated from the GI tract of rats [16] and was chosen for the present study mainly due to its strong ability to colonize and its anti-inflammatory effect in a colitis model [6, 17].

Biophotonic imaging (BPI) is a collection of non-destructive techniques that have greatly advanced studies of bacteria in different ecosystems. The recent development of the technology and increased diversity of BPI-related proteins, such as fluorescent and bioluminescence proteins, provide a wide variety of tools for biological imaging. Several versatile fluorescent reporter proteins are now available [18], with the red fluorescent protein mCherry being one of the most commonly used. It is one of the best choices for long-term imaging because of its high photo stability and its ease of detection under diverse *in vivo* and *in vitro* conditions where ultraviolet (UV) light can be used for detection [19]. Bioluminescence imaging (BLI) is a variant of BPI that can be used in a large variety of noninvasive *in vivo* and *in vitro* analytical applications. The luciferase reporter systems have been used in gene expression studies and for bioluminescence imaging. Luciferases comprise a large family of enzymes that catalyze the conversion of the substrate luciferin to oxyluciferin, which results in the emission of light. Several luciferase genes have been identified so far and the most commonly used utilize pathways that require oxygen, e.g. luciferases from firefly (*Photinus pyralis*) and click beetle luciferase (CBRed) from *Pyrophorus plagiophthalamus*. These require D-luciferin as a substrate, which in the presence of ATP is converted into the active luciferin [20].

Despite the existence of several reporter proteins, application of BPI to *Lactobacillus* species [21, 22] is largely undeveloped and so far there are no reports of a successful luminescence construct for *L*. *reuteri*. Probiotics of *Lactobacillus* are often orally administered and survive well on passage through the gastrointestinal tract. After introduction of the lactobacilli into a complex ecosystem such as the gut, keeping track of them is a challenge. Therefore, there is a limited knowledge about the fate of live probiotics after oral administration and of their interaction with the host and the intestinal microbiome. In addition, despite the recent explorations of probiotics-epithelial cross talk through specific pattern-recognition receptors (PRRs) [23, 24] or/and via certain receptor-ligand binding [10, 25] the precise mechanisms by which the health benefits are conferred are still far from being fully elucidated [26–28]. Therefore, generating this type of tool for *L*. *reuteri* could aid studies about their mechanisms of probiosis, localization and interactions with the host. It would also facilitate the development of new diagnostic applications for these reporter proteins in *in vitro* studies on *L*. *reuteri*, measuring e.g. gene expression, promoter strength, plasmid copy number and plasmid persistence.

Bacterial plasmids are widely used to introduce a reporter gene into a microorganism. Plasmids have diverse regulatory mechanisms for survival in a host [29] and play an important role in bacterial adaptability and diversity [30]. The ability of a plasmid to survive in a bacterial population in the absence of selection force is known as plasmid persistence. Plasmids with antibiotic resistance genes improve the fitness of their host, but in the absence of selection for the plasmid, persistence is a cost to host fitness. When introducing a new plasmid with a reporter gene, it is therefore important to evaluate its persistence with and without antibiotic pressure. Plasmid persistence in bacterial populations has previously been monitored in conventional studies using replica plating [31, 32]. However, the conventional culture-based method is labour-intensive, time-consuming and cannot be used in high-throughput screening. Thus, flow cytometry (FCM), real-time quantitative PCR (qPCR) and culture-based microscopy have recently been suggested as alternative methods for assessing plasmid persistence [33–36].

In this study, we report for the first time the construction and cloning of plasmids harbouring reporter genes expressing fluorescent (mCherry) and luminescent (CBRluc) proteins into *L*. *reuteri* strains ATCC PTA 6475 and R2LC, resulting in bacteria emitting strong signals of fluorescence and luminescence. The construct stability, plasmid persistence and signal intensity were studied by combining cultivation with microscopy, luminescence measurements and flow cytometry. We also examined whether flow cytometry could be an alternative to conventional plate count methods for routine measurement of plasmid persistence. As proof of concept the strains were detected by *in vivo* and *ex vivo* imaging. After intra-gastric administration of the strains to mice, bowel transit, localization and bacterial dose-dependent signal intensity were evaluated by an *in vivo* imaging system (IVIS). Finally, adhesion of fluorescent strains to cultured epithelial cells was studied by epifluorescent microscopy.

## Materials and Methods

### Animals

BALB/c male mice (Taconic Biosciences, Denmark) weighing between 18 and 35 g were used for *in vivo* imaging. The animals were kept under standardized conditions at 21–22°C and with daily illumination of 12/12 h darkness/light. All animal procedures were approved by the Swedish Laboratory Animal Ethical Committee in Uppsala and conducted in accordance with guidelines of the Swedish National Board for Laboratory Animals.

### Bacterial strains and growth conditions

The strains used in this study are listed in Table 1. The *Escherichia coli* strains were grown in Luria-Bertani agar and broth (Sigma Aldrich, Saint Louis, MI, USA) (1.5% NaCl was added to the LB) at 37°C with shaking at 150 rpm. The *L*. *reuteri* strains were cultured at 37°C in de Man Rogosa Sharpe (MRS) broth (Oxoid, Hampshire, UK) or under anaerobic conditions at 37°C on MRS agar (Oxoid, Hampshire, England). Strains harbouring pSIP411 or its derivatives and pCC1 vector were cultured in the presence of erythromycin (Sigma-Aldrich CHEMIE GmbH, Steinheim, Germany) if not otherwise stated. Erythromycin was added to the culture medium to a final concentration of 400 μg ml^-1^ for *E*. *coli* and 10 μg ml^-1^ for *L*. *reuteri*.

**Table 1:** Bacterial strains and plasmids used in this study.

Strains and plasmids	Description	Reference/Source
**Strains**		
*Lactococcus lactis*		
*Lactococcus lactis* MG1363		
*Lactobacillus reuteri*		
6475	ATCC PTA 6475 (previously designated MM4-1A). Wildtype, host strain. Human breast milk.	[77], A kind gift from BioGaia AB, Stockholm, Sweden.
R2LC	R2lC, Wildtype, Rat strain	[16], A kind gift from Siv Ahrné, Lund University, Sweden
6475-CBRluc-mCherry	6475 harbouring pSIP411-CBRluc-mCherry	This work
6475-mCherry	6475 harbouring pSIP411-mCherry	This work
R2LC-CBRluc	R2LC harbouring pSIP411-CBRluc	This work
R2LC-mCherry	R2LC harbouring pSIP411-mCherry	This work
*Escherichia coli*		
*E*. *coli* EPI300-pCC1-CBRluc-mCherry	Derivative of *E*. *coli* EPI300 harbouring pCC1-CBRluc-mCherry	Genscript, USA
*E*. *coli* PK401	Intermediate cloning host, dam^-^	[57]
**Plasmids**		
pCC1BAC^TM^	Chl^r^, trfA-based copy control vector with inducible promoter	Genscript, USA
pCC1BAC^TM^-*CBRluc-mCherry*	pCC1BAC^TM^ derivative containing CBRluc-mCherry under control of P11 (constitutive promoter)	This work
pJP059	A derivative of pNZ8048	[41], Kindly provided by Jan-Peter van Pijkeren, Michigan State University, USA
pSIP411	Em^r^; SppIP-based expression vector with P_SPPQ_::*gusA*	[40], Kindly provided by Lars Axelsson, Nofima, Norway
pSIP-*CBRluc-mCherry*	pSIP411 derivative containing *CBRluc-mCherry* under control of P_SPPQ_; Em^r^	This work
pSIP-*mCherry*	pSIP411 derivative containing *mCherry* under control of P_SPPQ_; Em^r^	This work
pSIP-*CBRluc*	pSIP411 derivative containing *CBRluc* under control of P_SPPQ_; Em^r^	This work

### Construction of plasmids and generation of *L*. *reuteri* recombinant strains

The plasmids used in this study are listed in Table 1 and the sequences in Fig A in S1 File. A dual-purpose CBRluc::mCherry cassette with distinct ribosomal binding sites for both reporter genes was designed. The 2492 bp cassette is flanked by *Bam*HI and *Xho*I sites and the genes are under control of the constitutive promoter P11 [37]. The cassette was codon-optimized for *Lactobacillus reuteri* and synthesized by GenScript (Piscataway, NJ, USA), which delivered it ligated into pCC1 cloned into *E*. *coli* EPI300. Two vectors based on the SH71 replicon, pSIP411 with the inducible PsppQ promoter (previously Porfx) and pJPO59, were used as cloning vectors [38]. *Escherichia coli* PK401 and *Lactococcus lactis* MG1363 were used as intermediate cloning hosts. *E*. *coli*, *L*. *lactis* and *L*. *reuteri* were transformed by electroporation using a GenePulser (Bio-Rad, Hercules, CA, USA) as described elsewhere [39, 40]. An electroporation buffer with 0.5% sucrose in 10% glycerol was used for *L*. *reuteri* strains [41]. Plasmid DNA was isolated using the QIAprep Miniprep kit (Qiagen, Hilden, Germany). The restriction enzymes were supplied by New England Biolabs (Ipswich, MA, USA) and the ligation steps were performed using T4 DNA ligase from Thermo Scientific (Carlsbad, CA, USA).

The following is a description of the cloning procedures: the CBRluc-mCherry fragment was excised from the pCC1 vector by *Nco*I-*Xho*I cleavage (the promoter region P11 flanked by *Bam*HI and *Nco*I was left in pCC1). In addition, pSIP411 was digested with *Nco*I and *Xho*I, resulting in excision of the *gusA* gene. The CBRluc-mCherry fragment was ligated into pSIP411 and the ligation mix transformed into *E*. *coli* PK401. Correct clones were identified by PCR and the pSIP-CBRluc-mCherry construct was isolated. It was then transformed into *L*. *reuteri* 6475 and the resulting strain was named 6475-CBRluc-mCherry.

In order to obtain constructs with separate reporter genes, the pSIP-CBRluc-mCherry construct was digested with different combinations of restriction enzymes. The CBRluc gene was removed by digestion with *Nco*I and *Sna*BI. Mung Bean nuclease was used to facilitate the subsequent blunt end ligation and the construct obtained was named pSIP-mCherry. Likewise, the mCherry gene was excised by digestion with *Mlu*I and *Xho*I. T4 DNA polymerase was used for 3´-overhang removal and 5´-overhang fill-in to form blunt ends. The construct obtained was named pSIP-CBRluc. The two new constructs were transformed into strains 6475 and R2LC by electroporation and named 6475-mCherry, R2LC-mCherry and R2LC-CBRluc. After each transformation, the inserted cassettes of expression vector were PCR-amplified and sequenced using Sip3m and Sip16 primers (Sip3m; 5´-CTAAGGAATTGTCAGATAGGC-3´ and Sip16; 5´-ATTAGTCTCGGACATTCTGC-3´).

The whole CBRluc::mCherry cassette under control of the constitutive promoter P11 was also inserted into pJPO59 cleaved with *Bam*HI and *Xho*I and the construct pJP059-CBRluc-mCherry was cloned into *Lactococcus lactis* MG1363 *and L*. *reuteri* 6475.

### Growth characteristics of recombinant strains

The dynamics of bacterial growth were monitored in MRS broth supplemented with erythromycin (10 μg ml^-1^). The growth of the recombinant strains 6475-CBRluc-mCherry, 6475-mCherry, R2LC-mCherry and R2LC-CBRluc was compared with that of the wildtype 6475 and R2LC in the presence (50 ng ml^-1^) and absence of SppIP inducing peptide (GenScript) [37]. The growth was evaluated as follows: *L*. *reuteri* wildtype and recombinant strains were cultivated overnight at 37°C. On the next day, the cultures were diluted to an optical density (OD_600_) of approximately 0.03 and 300 μl of culture were inoculated in triplicate wells of BioScreen polystyrene plates. The induction peptide SppIP (50 ng ml^-1^) was added after dilution and the plate was incubated for 24 hours at 37°C in a BioScreen C microbiology reader (Oy Growth Curves AB Ltd, Helsinki, Finland).

### Epifluorescence microscopy of the mCherry strains of *L*. *reuteri*

The mCherry production of *L*. *reuteri* strains was examined in liquid culture. The mCherry-producing strains were inoculated in MRS containing erythromycin and grown overnight at 37°C, after which 10 μl of culture were subcultured in 10 ml of medium daily over 10 days, obtaining approximately 100 generations in total. The signal intensity of mCherry-producing bacteria was evaluated at days 1, 4, 7 and 10 by visualization with fluorescence microscopy. On the day of evaluation of signal intensity, the overnight cultures were diluted to an OD_600_ of approximately 0.1 and incubated at 37°C until OD_600_ 0.2–0.3. Then, 50 ng ml^-1^ SppIP inducing peptide were added and the cultures were allowed to grow for four more hours. Samples with a longer induction period (20 hours) were also prepared and induction peptide was added at the time of inoculation. Four samples were prepared: bacteria in spent MRS broth with pH 4.6 and bacteria that had been washed and suspended in phosphate-buffered saline (PBS) with pH 7.2, both treated for 4 or 20 hours with 50 ng ml^-1^ SppIP inducing peptide. A drop of sample was placed on a glass slide and studied using a fluorescence microscope (Leica DMI 4000 inverted fluorescence microscope) equipped with a DFC360 FX (CCD) camera. The epifluorescence and light differential interference contrast (DIC; transmitted light) images were obtained using a HCX PL FLUOTAR 40x/0.60 CORR objective. The fluorescent bacteria were observed with a red light fluorescence filter (excitation: BP 515–560 nm, emission: Lp >590 nm). Images were taken and processed with the LAS AF Lite software (Leica). The images were presented as merged images (DIC/fluorescent (DIC/FLUO)).

The adherence of the mCherry producing bacteria was studied using a fluorescence microscope (Nikon Eclipse Ni-U Light Microscope (LM) coupled to a HGFI mercury lamp. The IPEC-J2 cells were stained with DAPI and incubated with mCherry expressing bacteria. The fluorescence and bright field images were observed at low magnification (40×) and images captured by a high-definition colour camera (DS-Fi2, Nikon, Japan). The fluorescent bacteria were observed with red light fluorescence filter (Epi-FL Filter set Texas Red, excitation 540–580 nm, emission 600–660 nm) and DAPI-labelled intestinal epithelial cell were observed through a blue GFP filter (excitation 360 nm, emission 460nm). The images were processed by Fiji image processing software (ImageJ/Fiji 1.46).

### Plate count assay

For the plate count assay, two biological replicates of 6475-CBRluc-mCherry, R2LC-CBRluc, 6475-mCherry and R2LC-mCherry were serially subcultured during 10 days and samples from days 1, 4, 7 and 10 were diluted and inoculated on primary plates (nonselective MRS agar). In order to screen for plasmid persistence, 100 colonies per strain were replica-plated from the primary plates to selective growth medium (MRS agar supplemented with erythromycin).

### Flow cytometry of mCherry-producing bacteria

For flow cytometry analysis, five biological replicates of the mCherry-producing bacterial strains 6475-CBRluc-mCherry, 6475-mCherry and R2LC-mCherry were serially subcultured during 10 days and induced as described above. The induced cells from days 1, 4, 7 and 10 of serial subculture were then harvested by centrifugation, washed with PBS and re-suspended in 10 ml PBS. The samples were analyzed in a FACScan Aria III cytometer (BD Biosciences, Franklin Lakes, NJ, USA) and a BD-LSR II cytometer (BD Biosciences) at the BioVis platform, SciLifeLab, Uppsala. In order to examine the effect of induction period on mCherry production, the cells were induced with SppIP (50 ng ml^-1^) directly after inoculation and allowed to grow overnight at 37°C. On the next day, the cultures were diluted to OD_600_ 1.5 and again induced with SppIP (50 ng ml^-1^) for 4 hours (total ~20 hours). Likewise, prior to flow cytometry assay, bacterial cell suspensions were prepared as described above. In order to measure fluorescence-expressing cells, the flow cytometer was set to 10 000–20 000 events for the different experiments. For each recombinant strain sample, PBS and wildtype strain were used as controls for proper gating and to measure background fluorescence. Samples were analyzed using the FACSDiva Version 6.0 software and dot plots of forward scatter (FSC) versus side scatter (SSC) of mCherry-producing bacteria were generated. A discriminatory gate was designated on each dot plot to include as many mCherry-positive cells as possible, while excluding as much debris and particles as possible. In addition, a histogram plot of cell counts versus fluorescent signal was generated where the fluorophores were excited with 561 nm laser and the emitted light detected with a 600 long pass (LP) and a 610/20 band pass (BP) filter under the same flow cytometry settings.

### Luciferase assay

The bioluminescence signal intensity of the recombinant strains was evaluated by inoculating the recombinant strains 6475-CBRluc-mCherry and R2LC-CBRluc in MRS broth and growing them in the presence of erythromycin overnight at 37°C. The cultures were serially subcultured every day for 10 days. Prior to submission of the bacterial cells producing CBRluc to the plate reader, the bacterial cells from days 1, 4, 7 and 10 of serial subculture were induced as described above for *in vitro* bioluminescence quantification. In parallel, non-induced cells were prepared similarly except for SppIP induction. The induced cultures were harvested by centrifugation at 5000 rpm for 10 min, washed in PBS and re-suspended again in PBS. The induced and non-induced bacterial samples (200 μl) were added in quadruplicate to a 96-well plate and 10 μl of 150 μg ml^-1^ XenoLight RediJect D-Luciferin (Caliper Life Sciences, Hopkinton, MA, USA) were added to each well. The signals were captured by a FLUOstar Omega plate reader (BMG Lab tech, Ortenberg, Germany) for 10 minutes after addition of D-Luciferin. The values obtained were expressed in relative light units (RLU) and normalized against the OD values of the bacterial cultures. The data were analyzed using the data analysis software MARS, linked with the Omega reader control software.

### *In vivo* and *ex vivo* imaging of *L*. *reuteri* strains in mice

The recombinant strains 6475-CBRluc-mCherry and R2LC-mCherry were grown, induced and harvested as explained in an earlier section. The bacterial cells were re-suspended and diluted in PBS and 15 μl induction peptide (75 ng ml^-1^) and 50 μl XenoLight RediJect D-Luciferin (30 mg ml^-1^) were added per ml of bacterial suspension. The bacterial dosages were very low (1×10^5^ CFU), low (1×10^6^ CFU), normal (1×10^8^ CFU) and high (1×10^10^ CFU) in a volume of 300 μl. Prior to the experiment, mice were anesthetized with 2% isoflurane. The bacterial mixtures were orally administered to mice intragastrically by gavage, where after the animals were placed in an IVIS Spectrum Pre-clinical *In Vivo* Imaging System (Caliper, PerkinElmer, Waltham, MA, USA) while 2% of isoflurane in air was administered through a nasal cone. To assess the transit of recombinant bacteria in GI tract, the *in vivo* images were taken at 0, 60, 120 and 180 minutes post gavage. Living-image software was used to generate pseudocolour images representing light intensity (ranging from dark blue designating the least intense to red designating the most intense). For *ex vivo* imaging, the GI tract was removed 0, 60, 120 and 180 min post gavage and directly imaged with the IVIS camera, without or with injection of air as described by others [42, 43]. The *in vivo* luminescent and fluorescent images were displayed in “photon” mode and the signal intensity was expressed as average radiance (p/sec/cm^2^/sr). The analyses were performed using the software Living Image (Caliper, PerkinElmer). We euthanized the mice by cervical dislocation while they were still under isoflurane anesthesia.

### IPEC-J2 Cells adherence assay

Adherence assays were performed on immobilized and suspended porcine jejunal epithelial IPEC-J2 cells (kindly provided by Nguyen Lien Thi Minh and Kerstin Skovgaard, Technical University of Denmark, National Veterinary Institute, Denmark). The assay on immobilized cells was performed as follows: approximately 3×10^5^ cells were seeded per well on a coverslip in a six-well tissue culture plate and grown and maintained in Dulbecco’s modified Eagle’s medium (DMEM)/ F-12 Ham containing 0.12% sodium bicarbonate, 15 mM HEPES, pyridoxine and L-glutamine (Sigma Aldrich), supplemented with Pen/Strep (Penicillin, 100 U ml^-1^ Streptomycin, 100 μg ml^-1^), 5% heat inactivated foetal bovine serum (FBS, Sigma Aldrich) and 0.5 mM sodium pyruvate and incubated at 37°C in 5% CO_2_ for 5 days. Medium was changed every other day. At day 5 of incubation, the medium was removed and non-adherent cells were washed away with Dulbecco`s Phosphate Buffered Saline (DPBS) three times. The mCherry producing bacteria, 6475-mCherry and R2LC-mCherry were grown and induced as described earlier. A bacterial count of 1×10^8^ CFU ml^-1^ was added to the intestinal cells in each well at the ratio 1000:1 (bacteria: IPEC-J2 cells) and incubated at 37°C in 5% CO_2_ for 1 hour. After incubation, the non-adherent cells were washed away with DPBS three times and the coverslip was stained with DAPI (AppliChem GmbH, Darmstadt, Germany) 1 μg ml^-1^ in methanol at 37°C for 15 minutes. Then the staining solution was removed and the coverslip washed once with methanol and dried upside down on a glass slide with a drop of glycerol as mounting medium. The prepared slides were kept in darkness at 4°C until examination by epifluorescence microscopy. We also performed an adhesion assay using suspensions of IPEC-J2 cells that were harvested by trypsinization and thereafter washed with DPBS. The bacteria were mixed with cells to achieve a bacterial to cell ratio of 1000:1 and incubated for one hour at 37°C. Thereafter, DAPI staining was performed as described above.

### Statistical analysis

Statistical differences in data from the flow cytometry analysis, luciferase assay and plate count were evaluated using two-way ANOVA and Tukey’s multiple comparisons post-hoc test. Differences were considered statistically significant at *p*≤0.05. All statistical analyses were performed with the JMP statistical software Pro11.

## Results

### Construction of bioluminescent and fluorescent *L*. *reuteri* strains and evaluation of their growth rate

**Fig 1:**
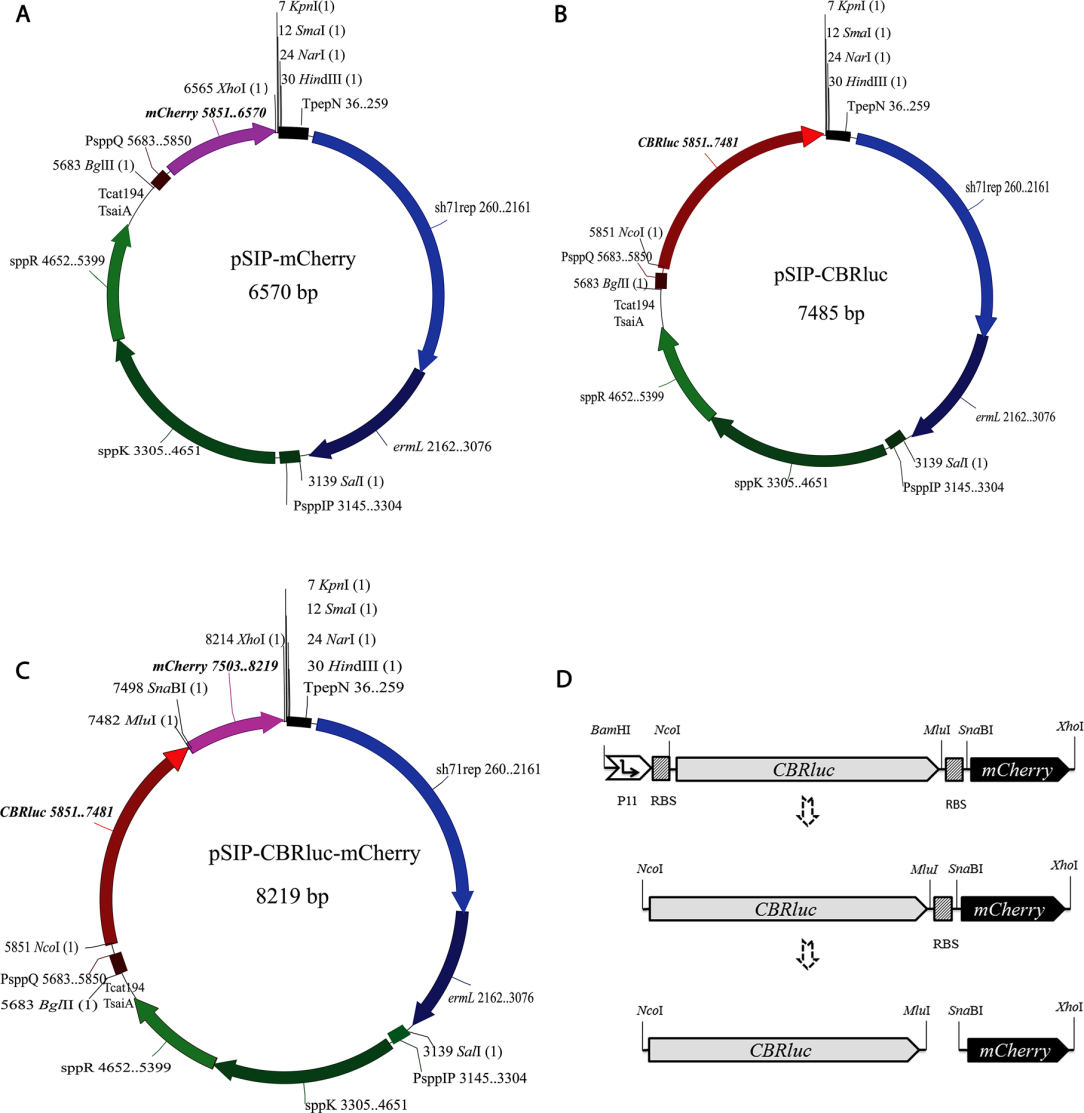
Schematic diagram of pSIP411 with inserted reporter genes. A. pSIP-CBRluc-mCherry. B. pSIP-CBRluc. C. pSIP-mCherry. The codon-optimized CBRluc-mCherry, CBRluc and mCherry fragments were cloned into the pSIP411 vector under control of the inducible promoter PsppQ, and the three constructs were introduced into *L*. *reuteri* 6475 and R2LC by electro-transformation. **D.** Map of the codon-optimized CBRluc::mCherry cassette under control of a constitutive promoter P11, and CBRluc and mCherry fragments prior to insertion into pSIP411.

Three codon-optimized reporter gene cassettes encoding click beetle red luciferase (CBRluc), red fluorescent protein mCherry and a united form, CBRluc-mCherry, were successfully inserted into the plasmid pSIP411 under control of the inducible promoter PsppQ [38]. The constructs were then transformed into the *L*. *reuteri* strains ATCC PTA 6475 and R2LC. Four out of six variants provided viable and correct clones and the resulting strains were named 6475-CBRluc-mCherry, 6475-mCherry, R2LC-mCherry and R2LC-CBRluc. The CBRluc::mCherry cassette was also ligated into pJP059 under control of the P11 constitutive promoter and the construct transformed into both *Lactococcus lactis* and *L*. *reuteri*. However, the construct was not stable and no functional clones could be obtained. The strains and details of plasmids constructed and used in this study are shown in Table 1 and Fig 1.

Factors affecting growth rate of the different strains, such as introduction of the recombinant vector, induction of protein expression and induction duration, were examined. The results showed that the 6475 strains had a higher growth rate than the R2LC strains. The growth of the recombinant strains in general was slightly impaired compared with the wildtype strains and the induction of the reporter genes further decreased the growth rate (Fig B in S1 File).

### Evaluation of plasmid persistence and signal intensity

**Fig 2:**
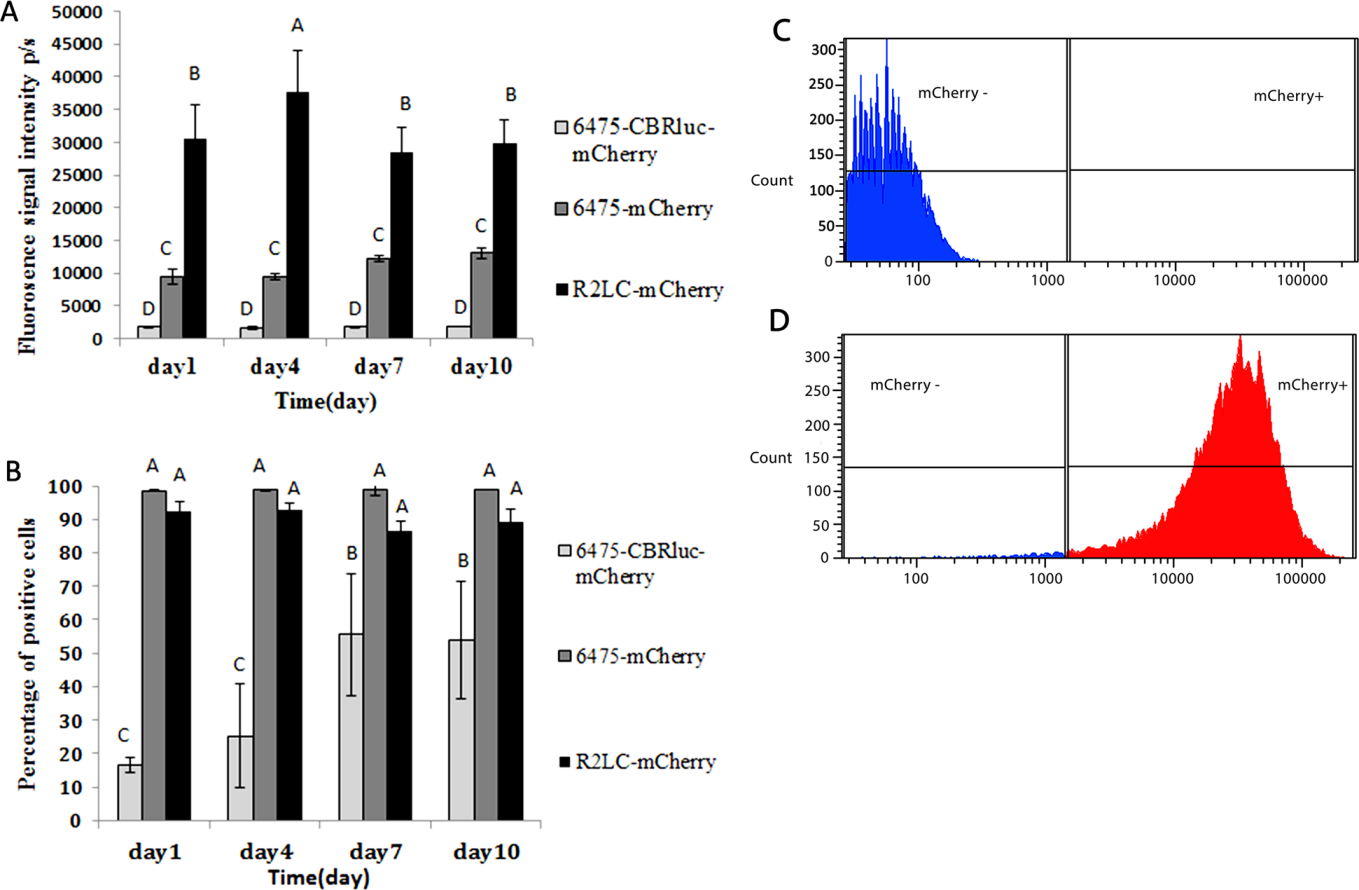
Flow cytometry analysis of mCherry-producing bacteria in the presence of selection pressure (erythromycin). **(A**) Percentage of fluorescent bacteria. (**B**) Fluorescence signal intensity of mCherry-expressing bacteria. (**C)** Flow cytometry population count histograms for 6475 wild type and (**D**) 6475-mCherry. mCherry—shown in blue and mCherry + in red. A fraction of plasmid-bearing cells from days 1, 4, 7, and 10 of the 10-day serial subculture in the presence of selection pressure were selected for monitoring signal intensity by FCM. For each recombinant strain sample, PBS and wildtype strain were used as control for initial SSC-FSC gates to measure background fluorescence. The error bars indicate the standard deviation values. Different letters above the columns indicate significant differences (*p*≤0.05).

**Fig 3:**
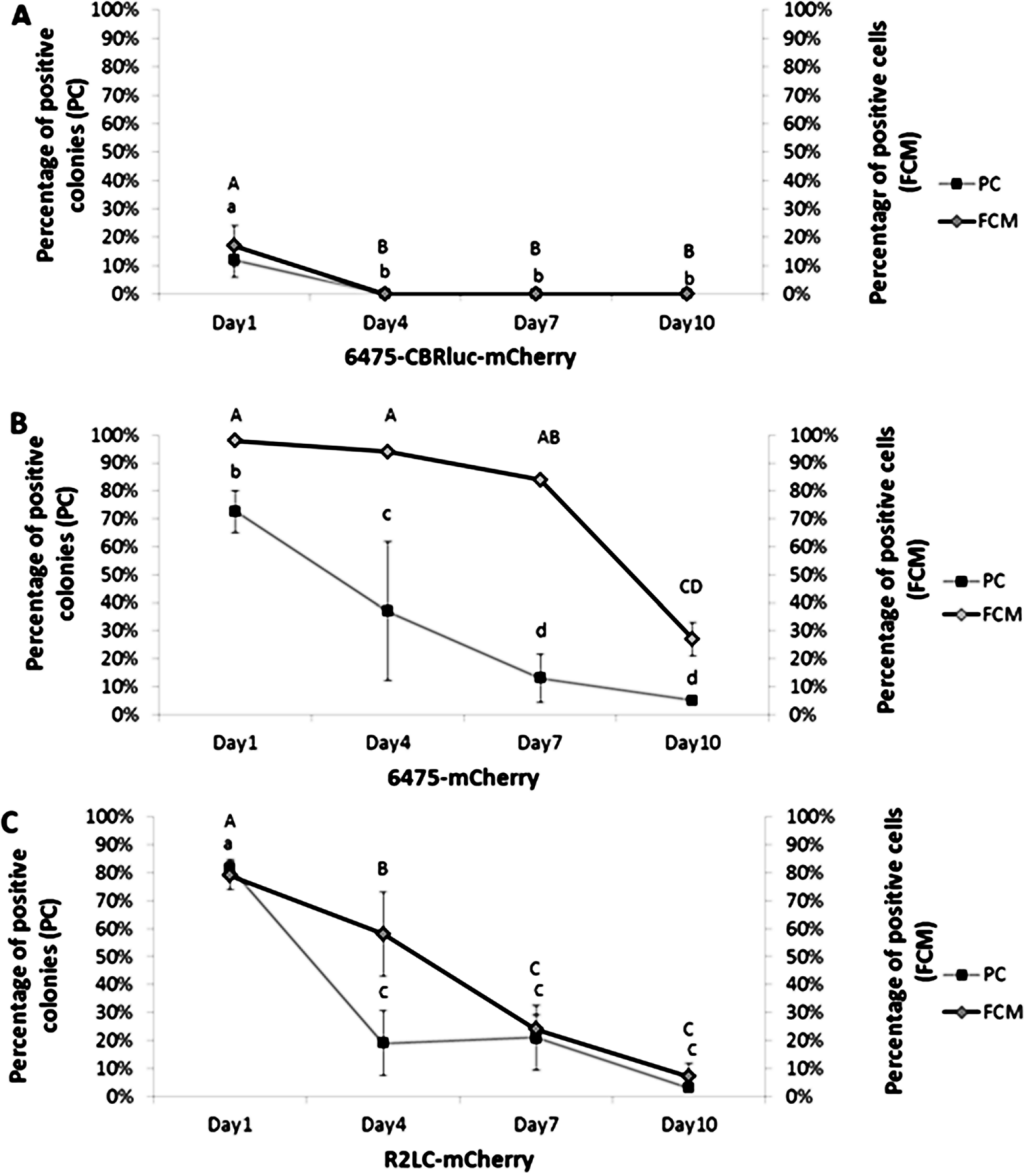
Plasmid persistence for each of the three mCherry-producing strains, measured by flow cytometry (FCM) and plate count (PC). Samples from days 1, 4, 7, 10 of a serial subculture in the absence of selective pressure (erythromycin) were analyzed. (**A**) 6475-CBRluc-mCherry. (**B**) 6475-mCherry. (**C**) R2LC- mCherry. The error bars show the standard deviation values obtained from five or three independent biological replicates. Different letters above the points indicate significant differences (*p*≤0.05).

Five biological replicates of 6475-CBRluc-mCherry, 6475-mCherry and R2LC-mCherry were cultivated in the absence of antibiotics. For flow cytometry (FCM) analysis, a histogram plot of cell counts versus fluorescent signal was generated as outlined above (Fig 2C and 2D). In parallel, plasmid persistence was evaluated by plate counts combined with replica plating (PC). The results from both the FCM and PC assays showed that in the absence of antibiotics, the proportion of plasmid-containing cells declined during subculture. The results from three PC assays showed that the percentage of R2LC-mCherry and 6475-mCherry still harbouring the constructs after 4 days of subculture were ~20 and ~ 35%, respectively. In comparison, FCM analysis of bacteria from day 4 showed plasmid stability of 60 and 95%, respectively (Fig 3B and 3C). The pSIP-CBRluc-mCherry plasmid had disappeared from 6475 at day 4 of serial subculture, as shown by both PC and FCM assays (Fig 3A). In contrast, in the presence of selection pressure, observations from fluorescent microscopy (FLM) and FCM indicated that signal intensity was stable over a period of 10 days of daily subculture of the bacteria (Fig 2B and data not shown).

**Fig 4:**
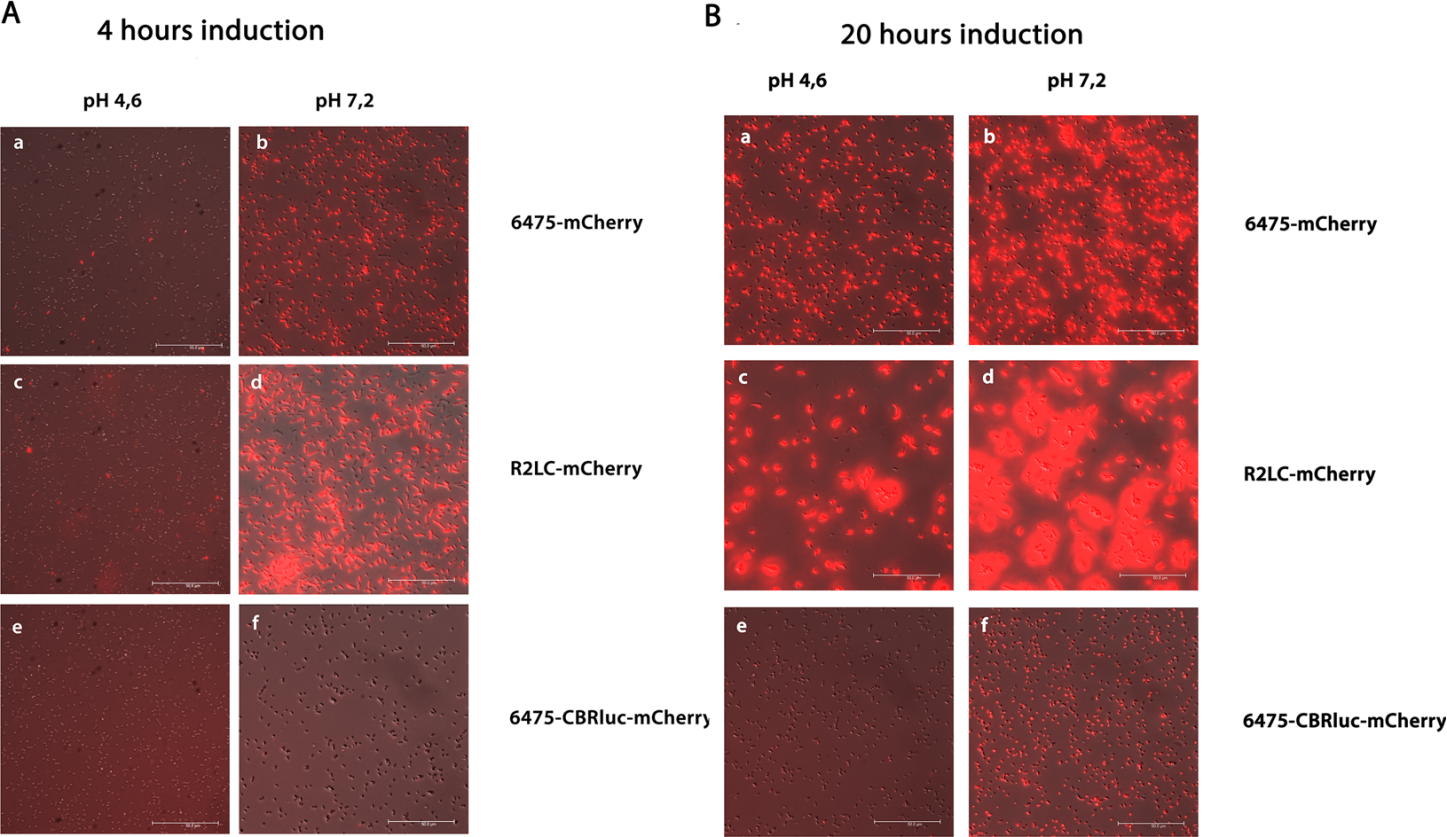
Epifluorescence images (light differential interference contrast (DIC)/Fluorescent (DIC/FLUO)) of *L*. *reuteri* strains 6475-mCherry, 6475-CBRluc-mCherry and R2LC-mCherry. (**A**) Images of recombinant bacteria after 4 hours of induction. (**B**) Images of recombinant bacteria after 20 hours of induction. The effect of pH and signal intensity on signal intensity was analyzed. The fluorescence signal from cells with long or short induction time (~4 vs ~20 hours) that had been cultured in MRS broth with a final pH of 4.6 was compared with that from cells at pH 7.2 under red light fluorescence filter (40x). (**A and B): (a, b)** 6475-mCherry; (**c, d)** 6475-CBRluc-mCherry; (**e, f)** R2LC-mCherry. All the images are presented as merged (DIC/FLUO). The scale bar shows 50 μm.

6475-CBRluc-mCherry and R2LC-mCherry exhibited the lowest and highest (p<0.001) signal intensity among the three fluorescent strains, with a median value of 1.8×10^3^ and 2.9 ×10^4^ p/s, respectively (Fig 2A). This was confirmed by microscopy (Fig 4). However, microscopy showed different degrees of signal heterogeneity among plasmid-bearing cells (Fig 4). Besides, cultivation in the presence of selection pressure showed that close to 100% of 6475-mCherry and R2LC-mCherry cells were positive, but that only 17% of the 6475-CBRluc-mCherry cells produced detectable amounts of fluorescence. However, the percentage increased (*p*<0.01), to 56%, after 10 days of subculture (Fig 2B).

**Fig 5:**
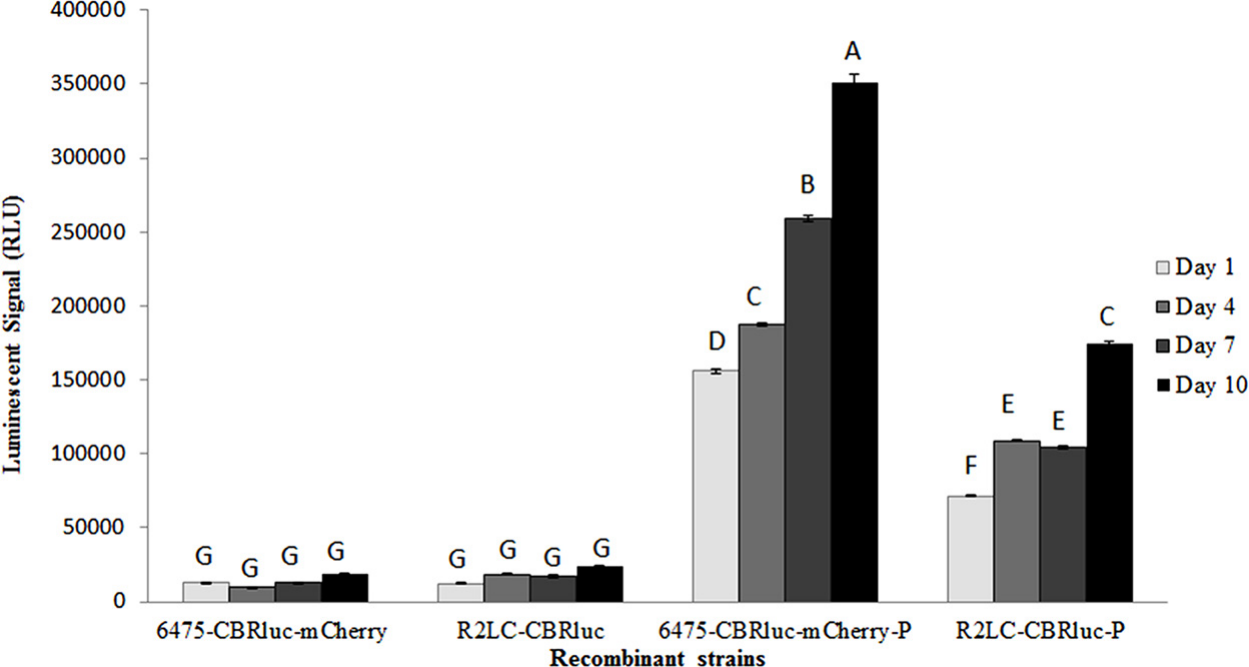
Bioluminescence assay of induced and non-induced *L*. *reuteri* 6475-CBRluc-mCherry and R2LC-CBRluc. The signal stability was examined during 10 days of subculture using luciferase assay. The signal intensity of the two strains was analyzed both in the presence and absence of induction peptide (P). Differences in signal intensities were analyzed by ANOVA and Tukey’s post-hoc test. Columns labelled with different letters are significantly different (p≤0.05).

The production of bioluminescence by 6475-CBRluc-mCherry and R2LC-CBRluc was analyzed using a luciferase assay. Samples taken after 1, 4, 7 and 10 days of serial subculture in the presence of selection pressure were analyzed. The luciferase signal intensity of both strains increased and had almost doubled at day 10 of subculture (Fig 5). In addition, weak background expression (without induction) could be detected. Stability of the plasmids harbouring the luciferase gene was studied by PC. This showed that the percentage of the R2LC-CBRluc cells harbouring the plasmid was higher than the percentage of 6475-CBRluc-mCherry (77% at day 1 and 7% at day 10 compared with 12% and 0%, respectively) Fig C in S1 File.

### Improvement of mCherry signal

Influences of pH on mCherry signal and induction duration on mCherry expression were studied using both FLM and FCM. Microscopy analysis indicated a clear effect of pH on the fluorescence signal intensity and improvement of signals from mCherry-expressing bacteria was observed at pH 7.2 compared with pH 4.6 (Fig 4).

**Fig 6:**
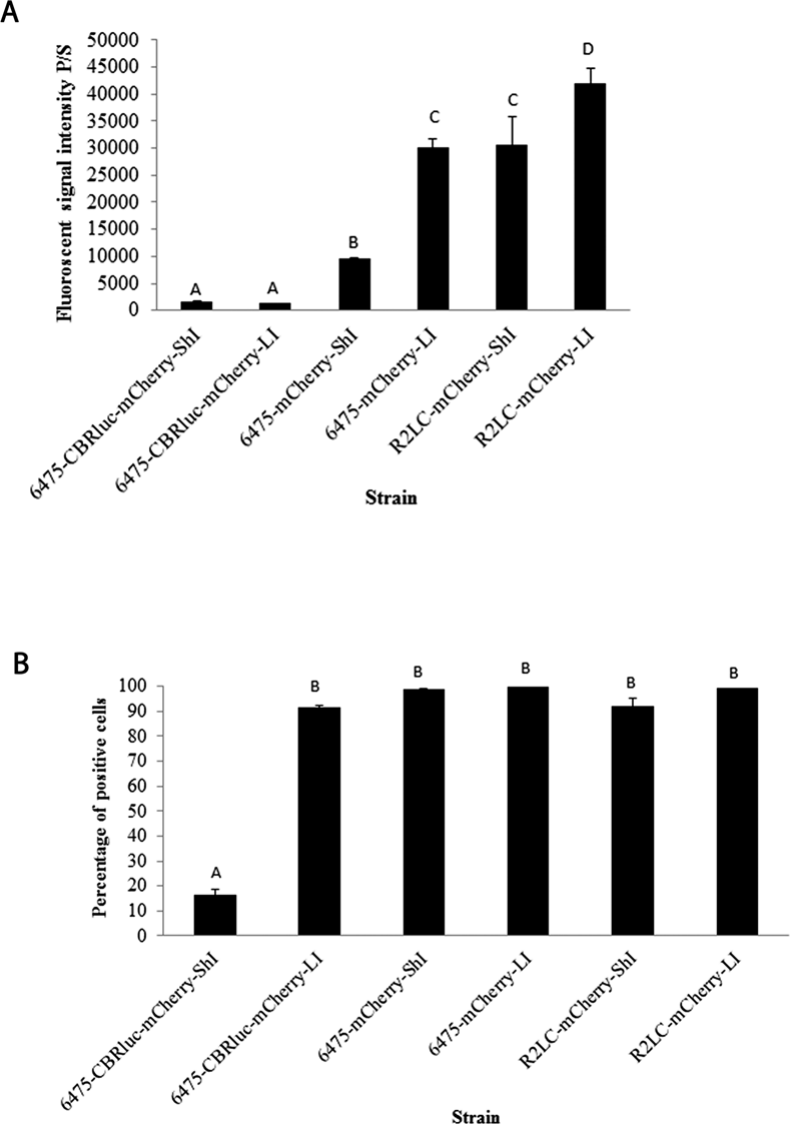
Combined effect of induction duration and subculture on mCherry-producing strains. Bacterial cultures in the presence of antibiotics were induced and a fraction of cells analyzed by flow cytometry after a short induction (ShI) (~4 hours) or long induction (LI) (~20 hours) period. Columns labelled with different letters are significantly different (*p*≤0.05). The error bars indicate the standard deviation of median values obtained from five independent biological replicates.

In addition, the signal intensity was significantly (*p<*0.001) improved with a long induction (LI) period (~20 hours) compared with a short induction (ShI) period (~4 hours) for the mCherry-producing strains 6475-mCherry (9.5×10^3^ p/s and 3×10^4^ p/s, respectively) and R2LC-mCherry (3×10^4^ and 4.2×10^4^, respectively), as measured by FCM and also shown by FLM (Fig 6A, Fig 4). We also found that the percentage of fluorescent 6475-mCherry and of R2LC-mCherry cells was close to 100% (Fig 6B). On the other hand, the percentage of fluorescent 6475-CBRluc-mCherry was considerably lower, but besides the above-mentioned increase after subculture of the strain (Fig 2A), the percentage also increased (*p≤*0.01) with a long induction period (day 1 from 17% to 92% and day 7 from 56% to 84% Fig D in S1 File. This was also confirmed by FLM (Fig 4A and 4B).

### Intravital imaging of luminescent and fluorescent *L*. *reuteri*

**Fig 7:**
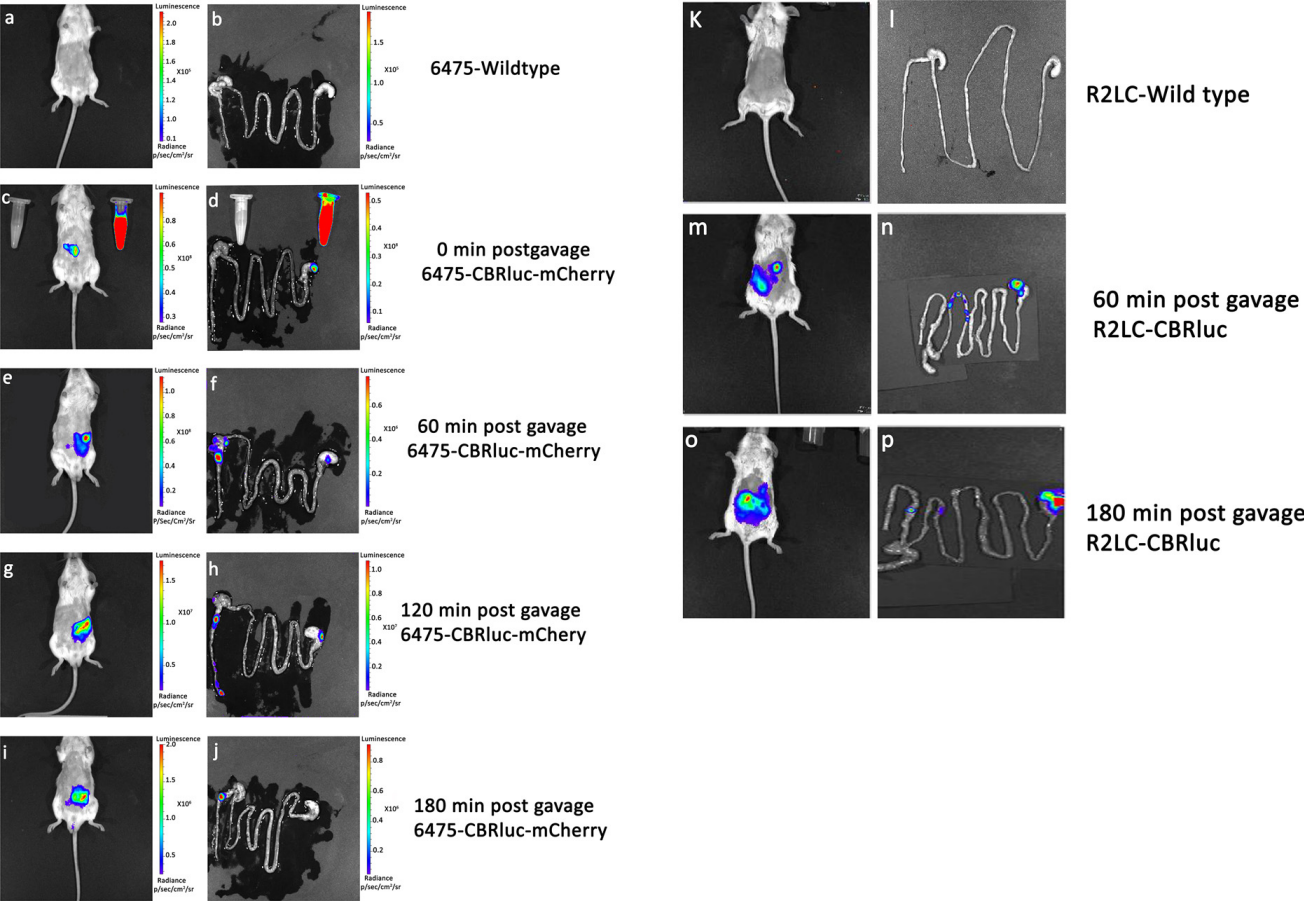
Gastrointestinal transit of luminescent *L*. *reuteri* strains. Localization of 6475-CBRluc-mCherry, R2LC-CBRluc and wildtype strains was evaluated *in vivo* and *ex vivo* using IVIS at 0, 60, 120 and 180 min after administration of a single dose of the bacteria (1x10^10^ CFU/mouse) in 3 separate experiments. 6475-CBRluc-mCherry (n = 10), R2LC-CBRluc (n = 3), 6475-wild type (n = 3) and R2LC-wild type (n = 1) for luminescence and 6475-CBRluc-mCherry (n = 5) and 6475-wild type (n = 2) for fluorescence imaging. 6475-wildtype (**a, b**); 6475-CBRluc-mCherry 0 min (**c, d**); 60 min (**e, f**); 120 min (**g, h**); and 180 min post gavage (**i, j**); R2LC-wildtype (**k, l**); R2LC-CBRluc 60 min (**m, n**) and 180 min post gavage (**o, p**).

**Fig 8:**
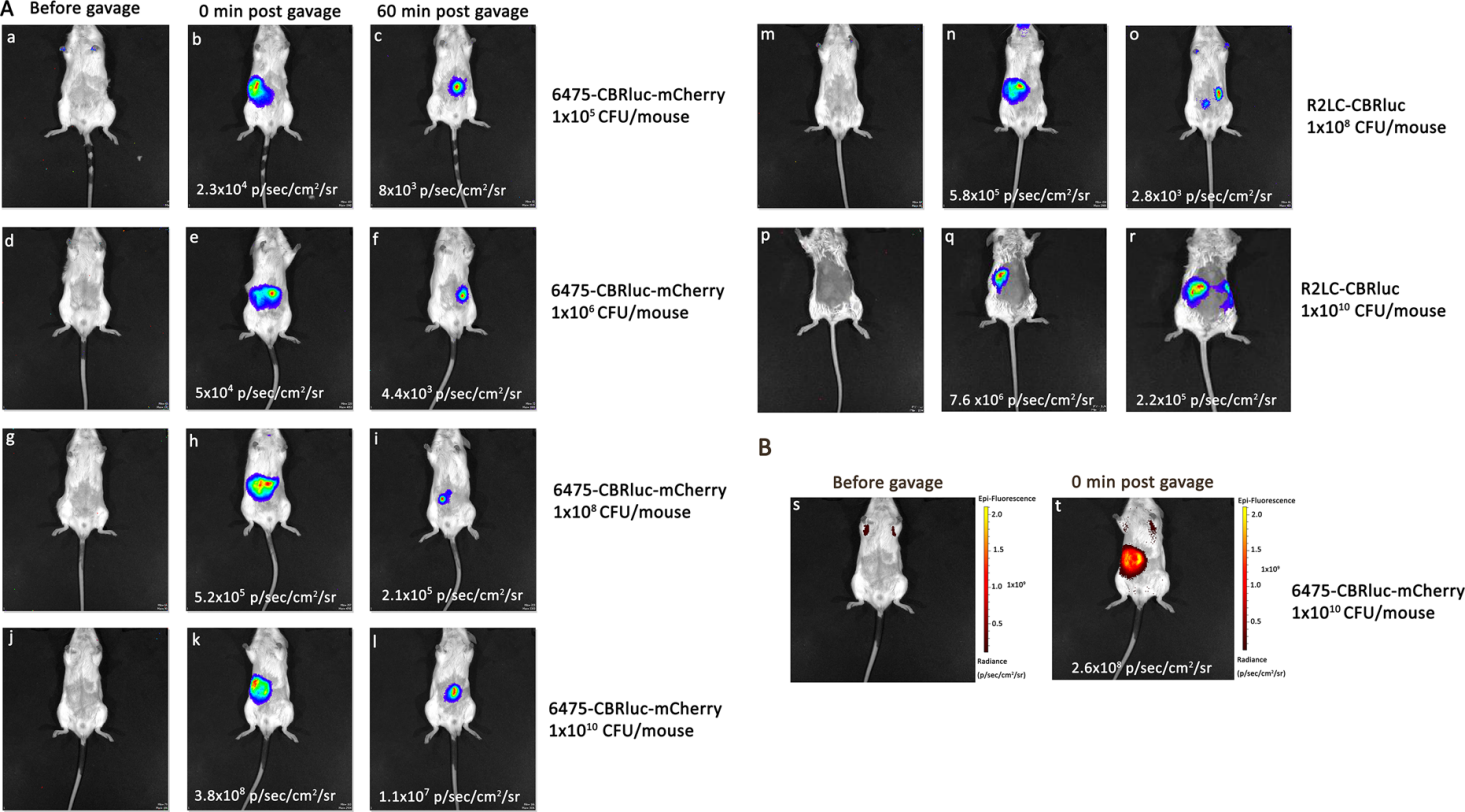
*In vivo* biophotonic imaging of 6475-CBRluc-mCherry and R2LC-CBRluc given at different doses. In 3 separate experiments for luminescence and fluorescent imaging, BALB/c male mice were gavaged with fluorescent and luminescent strains: 6475-CBRluc-mCherry (n = 10), R2LC-CBRluc (n = 3) for luminescence and 6475-CBRluc-mCherry (n = 5) for fluorescence imaging. The signal intensities were compared 0 and 60 min after an intra-gastric inoculation. (**A**) Bioluminescence imaging of: (**a, b, c**) 6475-CBRluc-mCherry, 1x10^5^ CFU/mouse; (**d, e, f**) 6475-CBRluc-mCherry, 1x10^6^ CFU/mouse; (**g, h, i**) 6475-CBRluc-mCherry, 1x10^8^ CFU/mouse; (**j, k, l**) 6475-CBRluc-mCherry, 1x10^10^ CFU/mouse; (**m, n, o**) R2LC-CBRluc, 1x10^8^ CFU/mouse; (**p, q, r**) R2LC-CBRluc, 1x10^10^ CFU/mouse. (**B**) *In vivo* fluorescence imaging of: (**s, t**) 6475-CBRluc-mCherry, 1x10^10^ CFU/mouse before and immediately post gavage.

*In vivo* and *ex vivo* quantification and visualization of the luminescence and fluorescence producing strains were performed after administration of different doses of the bacteria (from 1×10^5^ to 1×10^10^ CFU/mouse). The background levels of luminescence and fluorescence were estimated for mice that had been orally inoculated with a wild type strain (Fig 7A and 7B) and/or prior to intra-gastric administration of 6475-CBRluc-mCherry and R2LC-CBRluc by IVIS imaging (Fig 8A and 8B). The highest luminescence signals were obtained directly after gavage, 3.8×10^8^ and 7.6×10^6^ p/sec/cm^2^/sr, from mice that had been inoculated with the high doses of 6475-CBRluc-mCherry and R2LC-CBRluc, respectively (Fig 8A). Compared with this, giving a 100 fold lower dose (normal dose), 6475-CBRluc-mCherry and R2LC-CBRluc delivered 5.2 x10^5^ and 5.8x10^5^ p/sec/cm^2^/sr, respectively (Fig 8A). BPI allowed us also to observe the lowest dose of 6475-CBRluc-mCherry (1×10^5^ CFU/mouse, Fig 8A). At 60 min post gavage, a reduction in the signals was seen for all doses tested (Fig 8A). However, at 3 hours post gavage, the bioluminescence signals of mice given the high dose were still strong when measured *ex vivo* (Fig 7A and 7B).

The fluorescence producing 6475-CBRluc-mCherry could also be monitored *in vivo*. The highest dose (1×10^10^ CFU/mouse) gave detectable signals directly post gavage, but signals could not be detected after 60 min (Fig 8B).

The bowel transit and localization of luminescent 6475-CBRluc-mCherry and R2LC-CBRluc were compared and monitored *in vivo* and *ex vivo* using IVIS. Directly post gavage, both strains were detected in the stomach, 1 hour post gavage signals were received from stomach, ileum and caecum for 6475-CBRluc-mCherry and from the stomach and ileum for R2LC-CBRluc, and 3 hours post gavage R2LC-CBRluc still gave strong signals from the stomach whereas 6475-CBRluc-mCherry only could be detected in the colon (Fig 7A and 7B).

### Adhesion of mCherry-producing bacteria to an intestinal epithelial cell line

**Fig 9:**
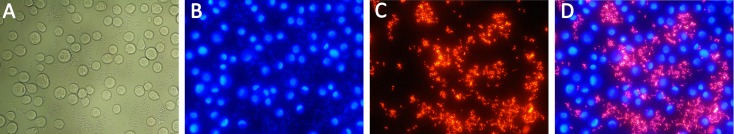
Fluorescence microscopy imaging of R2LC-mCherry adhering to IPEC-J2 cells. (A) Bright-field image. (**B**) Fluorescence imaging of the DAPI stained IPEC-J2 cells. (**C**) Fluorescence imaging of R2LC-mCherry. (**D**) Merging of the B and C images.

mCherry-producing R2LC-mCherry was evaluated in an adhesion assay using the porcine epithelial cell line IPEC-J2. Both immobilized cells grown on cover slips and cells in suspension were used and the ratio between bacteria and epithelial cells was 1000:1. In both experiments large amounts of bacteria adhered efficiently to the epithelial cells. In fact, using immobilized cells it was observed that most of the bacteria adhered to the intracellular junctions between neighbouring cells (Fig 9).

## Discussion

Development of bioluminescent and fluorescent *L*. *reuteri* is an important step towards improving the possibilities for *in vivo* and *in vitro* studies of interactions between this probiotic species and the host. Therefore, codon-optimized genes encoding CBRluc and mCherry were designed, inserted into the expression vector pSIP411 and transformed into two strains of *L*. *reuteri*.

### *In vitro* evaluation of plasmid persistence and signal intensity

We investigated whether flow cytometry (FCM) could be an alternative to the conventional plate count (PC) method for measurement of plasmid persistence. The plasmid stability profile obtained by both FCM and PC indicated that the recombinant plasmids (pSIP-CBRluc-mCherry, pSIP-CBRluc and pSIP-mCherry) in non-selective cultures were not entirely stable and the number of plasmid-bearing bacteria was dramatically decreased after being subcultured for 10 days (corresponding to approximately 100 generations; Fig 3). As an example, the stability data for 6475-CBRluc-mCherry generated by both FCM and PC were similar and indicated 100% plasmid loss at day 4 (Fig 3A). Loftie-Eaton et al. have also found that the plasmid persistence profiles generated by these techniques are similar [34]. However, the plasmid loss rate of 6475- mCherry detected by PC was clearly higher than the rate shown by FCM.

In addition, PC often gave a vague result, with pronounced differences in the number, size and form of positive colonies. This could possibly be explained by variations in growth rates between plasmid-containing and plasmid-free cells, while the dominant plasmid-containing cells accumulate in the culture [44]. These ambiguous results, with differing growth rates and possible underestimation of the number of plasmid-containing cells identified by PC, might be caused by insufficient plasmid segregation [45–47], leading to a decrease in plasmid copy number in a certain population of plasmid-bearing bacterial cells. Hence, the values obtained could vary due to limitations of the plate count assay in precise measurement of plasmid persistence in cases where the cells harbour very low copy number plasmids. Moreover, the PC method does not scale well for high-throughput applications. In contrast, FCM is a powerful analytical technique for analyzing bacteria with low fluorescence signal intensity in a complex population within a short period. Moreover, in contrast to PC, FCM is a scalable visualization technique for representing large-scale data and a selectable marker is not actually a required element for monitoring the plasmid persistence by FCM. However, a fluorescent reporter gene is needed to assess the presence or absence of plasmid in bacterial populations, and biased counting due to the existence of autofluorescence within the cells could be a drawback.

Both FLM and FCM showed that the fluorescence signal intensity did not decrease over a period of 10 days of serial subculture in the presence of selection pressure. In fact, the observations during 10 days of serial subculture showed a significant improvement in the fluorescence signal intensity for one of the strains, 6475-CBRluc-mCherry (Fig 2A). A similar result was observed in the luciferase assay, where 6475-CBRluc-mCherry displayed approximately double the luciferase expression seen in R2LC-CBRluc and, interestingly, the luciferase signal in both strains doubled from day 1 to 10 (Fig 5). Possible explanations for this improvement of signal intensity are an increase in the number of plasmid-containing cells, whereby the plasmid–host adaptation dynamics are improved as a result of clonal interference [48, 49], or that more cells produced a detectable amount of signal.

We demonstrated that selection force is necessary for long-term persistence of the plasmids used and that plasmid persistence imposes a fitness cost on the host, which is a necessary investment. However, it is possible that during selection pressure, the introduction of mutations in the host or the plasmids might have increased the adaptation of the plasmids in the *L*. *reuteri* hosts. Sota et al. showed that the stability of the plasmid pMS0506 significantly improved after 200 generations and that after 1000 generations, one or two genotypes dominated the populations [50]. This improvement could be explained by cost amelioration in the host through introducing mutations in the plasmid, leading to adaptation to a new host. A number of studies have shown that lack of plasmid persistence can be improved by adaptation of the plasmid through cost amelioration [48, 51], in particular mutations of the plasmid [50, 52, 53]. Host chromosomal mutation is another factor that can play a significant role in plasmid-host adaptation and increase host fitness [52, 54].

There might also be a correlation between the size of the reporter genes and plasmid persistence, as plasmids with a smaller insert appeared to be more stable. The largest plasmid, pSIP-CBRluc-mCherry, showed the lowest persistence of the three plasmids and none of the methods could detect the plasmid after 4 days of serial subculture without selection pressure (Fig 3). It has previously been shown that plasmid size is negatively linked to plasmid copy number [55]. Others have shown that the feature of the insert sequence could be the reason for the weak plasmid persistence [55, 56].

We also found that while both R2LC-mCherry and 6475-mCherry had close to 100% positive cells in the presence of selection pressure, R2LC-mCherry had stronger signal intensity as shown by FCM analysis (Figs 2B and 4). This indicates that the amount of fluorescent protein produced per cell was higher for R2LC-mCherry than for 6475-mCherry, which might be a result of higher plasmid copy numbers and/or higher promoter activity in this strain. Indeed, pSIP411 appears to be a multi-copy-number plasmid (multicopy plasmids are structurally [57] and segregationally [58] unstable) and is reported to have nine copies when transformed into *L*. *sakei* Lb790 and approximately 48 in *L*. *plantarum* NC8 [38]. It could possibly also have different copy numbers in different *L*. *reuteri* strains.

### Improving the fluorescence signal from mCherry-producing *L*. *reuteri*

The fluorescence signal intensity of mCherry-producing strains in spent MRS broth (pH 4.6) was improved when they were moved to a buffer with neutral pH. Sensitivity of mCherry to acidity has been reported previously by Shaner et al. [59]. Apart from pH [60, 61], the fluorescence intensity strongly depends on the maturation time of mCherry and it has been shown that it can be affected by several factors including oxygen availability [62, 63] temperature [63, 64], species [65, 66] and even strain of bacteria [67].

In addition, the expression of mCherry protein from all mCherry-producing strains increased with duration of incubation with the SppIP inducing peptide. This could possibly be explained by a combination of longer induction and maturation time. Flow cytometry analysis of 6475-CBRluc-mCherry showed a clear increase in the percentage of positive cells after a long compared with a short induction period (Fig 6) and a similar increase was seen when measuring the luminescence. Besides development of plasmid-host adaptation, the reason for this could be that many positive cells had undetectable signals after a short induction period, but the signal intensity pushed above the detection limit after a long induction period.

We also observed that the expression of reporter protein was not limited to induced bacteria and non-induced bacterial cells had a very low expression of mCherry according to fluorescent microscopy (data not shown) and CBRluc (as shown in Fig 5). This production could be due to promoter leakage of pSIP411 plasmid, which has also been observed by Sørvig et al. [38].

### Intravital imaging of luminescent and fluorescent *L*. *reuteri*

Both luminescent and fluorescent *L*. *reuteri* were monitored using intravital imaging and a dose as low as 10^5^ luminescent bacteria per mouse could be detected. There are no other reports of bioluminescence *in vivo* imaging for *L*. *reuteri*, but recently a successful attempt to visualize CBRluc producing *L*. *plantarum* NCIMB8826 and *Lactococcus lactis* MG1363 by *in vivo* bioluminescence imaging in mice was reported by Daniel et al. [21]. The signal production and transit of bioluminescence bacteria given at 5×10^10^ CFU/mouse were studied, and it proved possible to detect a bioluminescent signal of (~3 × 10^11^ p/s) for *L*. *plantarum*-CBRluc, which was approximately 100-fold higher than that of *L*. *lactis*-CBRluc [21]. In addition, *in vivo* imaging of *L*. *lactis* MG1363 expressing the *luc* operon has been reported by Lee et al [68]. A maximum signal of 7.31×10^5^ p/sec/cm^2^/sr was detected directly after oral administration of *L*. *lactis* MG1363 (pMG36e_luc+) (~ 1×10^9^ CFU/rat), although bioluminescence signals were not detected 3 hours post gavage [68]. In the present study, a bioluminescence signal of 2.1×10^8^ p/sec/cm^2^/sr (~ 1×10^10^ CFU/mouse) post gavage was detected by whole body imaging.

The transit and localization of the luminescent strains 6475-CBRluc-mCherry and R2LC-CBRluc were compared after administration of a single dose of the bacteria. The bacterial transit time for the two recombinant strains varied. R2LC-CBRluc showed a slower transit and was found in the ileum 60 min post gavage, while 6475-CBRluc-mCherry was found in the caecum and colon. However, large amounts of the luminescent R2LC were found in the stomach 3 hours post gavage, possibly as a result of a niche specificity of this strain. Lactobacilli often colonise the stratified squamous epithelium of the forestomach of rodents. This has previously been shown to be a feature of rodent strain R2LC, but not of *L*. *reuteri* originating from humans [69, 70].

Similarly to Bourgois et al. [71] and others [20], we observed that the luciferase production was improved in the presence of oxygen, required as a co-factor for the luciferase reaction. However, the need for oxygen is low and an oxygen concentration as low as 10 nM is enough for detection of luminescence in murine GI tract [71]. We performed a comparison study on fed and starved mice which indicated that the presence of food in the stomach was required to retain the oxygen and we succeeded in performing the imaging without air injection in fed animals, although the signal intensity declined slightly without air injection.

Finally, we studied adhesion of mCherry expressing strains to IPEC-J2 cells and observed that a large proportion of the bacteria adhered to intra cellular junctions of the epithelial cells. Many bacteria adhere to host cell surface structures, often with a preference for the junctions between the cells. Pathogens such as *Listeria monocytogenes* and *Helicobacter pylori* invade epithelial cells by interacting with the apical-junctional complex, either by binding to cell adhesion molecules (CAMs) such as E-cadherin, causing rearrangement of the actin cytoskeleton [72, 73], or through modulating the tight-junction proteins, leading to destabilization of the junctional machinery and penetration of the bacteria across the host epithelial junctional barrier [74]. Probiotic bacteria have also been shown to interact with the junctional complex, but in contrast to pathogens they have been shown to up-regulate the expression of either adherence junction [75] or tight junction proteins [76], which reinforce the barrier function of the epithelial cells.

In conclusion, we have demonstrated that the two reporter genes are suitable markers for studying *L*. *reuteri* in the GI tract and the application of fluorescence-expressing bacteria for high-throughput screening of plasmid persistence using FCM. However, the application of mCherry and luciferase-labelled lactobacilli are not limited to study the bacterial transit and persistency. They also have significant potential of *in vivo* and *in vitro* studies of the interactions between lactobacilli and host’s cells and structures.

## Supporting Information

S1 File Fig A. Nucleotide sequence and corresponding amino acid sequence of the CBRluc-mCherry cassette. The whole cassette, including the constitutive promoter (P11), is 2492 bp. The P11 fragment flanked by *Bam*HI and *Nco*I is 123 bp, the CBRluc fragment flanked by *Nco*I and *Mlu*I is 1631 bp and the mCherry fragment flanked by *Sna*BI and *Xho*I is 714 bp. Features of the sequence are specified as follows: nucleotide sequence with underlined capital letters = P11 promoter region; bold underlined nucleotide sequence with capital letters = ribosomal binding sites; italic letters = restriction enzyme cleavage sites. Fig B. Growth curves of recombinant and wildtype strains. The growth of 6475-CBRluc-mCherry, 6475-mCherry, R2LC-mCherry and R2LC-CBRluc was compared with that of wildtype strains ATCC PTA 6475 and R2LC in the presence or absence of SppIP inducing peptide. Fig C. Measurement of plasmid stability using replica plating. 6475-CBRluc-mCherry and R2LC-CBRluc were cultured serially in MRS broth without selection pressure for 10 days. Samples from days 1, 4, 7 and 10 were replica-plated to evaluate the plasmid persistence. Fig D. Combined effect of induction period and subculture on mCherry-producing strains. **(A)** Fluorescence signal intensity. **(B)** Percentage of mCherry-producing bacteria. Samples from days 1 and 7 of a serial subculture in the presence of antibiotics were analyzed by flow cytometry after a short or long induction period. Columns labelled with different letters are significantly different (p≤0.05). The error bars indicate the standard deviation of median values obtained from five independent biological replicates.
(DOCX)
