# Supplementary material for: In Vivo and In Vitro Detection of Luminescent and Fluorescent Lactobacillus reuteri and Application of Red Fluorescent mCherry for Assessing Plasmid Persistence
Source: PLoS One. 2016 Mar 22;11(3):e0151969. doi: 10.1371/journal.pone.0151969 (PMC4803345; doi:10.1371/journal.pone.0151969)
Supplement: S1 File — Fig A. Nucleotide sequence and corresponding amino acid sequence of the CBRluc-mCherry cassette. The whole cassette, including the constitutive promoter (P11), is 2492 bp. The P11 fragment flanked by BamHI and NcoI is 123 bp, the CBRluc fragment flanked by NcoI and MluI is 1631 bp and the mCherry fragment flanked by SnaBI and XhoI is 714 bp. Features of the sequence are specified as follows: nucleotide sequence with underlined capital letters = P11 promoter region; bold underlined nucleotide sequence with capital letters = ribosomal binding sites; italic letters = restriction enzyme cleavage sites. Fig B. Growth curves of recombinant and wildtype strains. The growth of 6475-CBRluc-mCherry, 6475-mCherry, R2LC-mCherry and R2LC-CBRluc was compared with that of wildtype strains ATCC PTA 6475 and R2LC in the presence or absence of SppIP inducing peptide. Fig C. Measurement of plasmid stability using replica plating. 6475-CBRluc-mCherry and R2LC-CBRluc were cultured serially in MRS broth without selection pressure for 10 days. Samples from days 1, 4, 7 and 10 were replica-plated to evaluate the plasmid persistence. Fig D. Combined effect of induction period and subculture on mCherry-producing strains. (A) Fluorescence signal intensity. (B) Percentage of mCherry-producing bacteria. Samples from days 1 and 7 of a serial subculture in the presence of antibiotics were analyzed by flow cytometry after a short or long induction period. Columns labelled with different letters are significantly different (p≤0.05). The error bars indicate the standard deviation of median values obtained from five independent biological replicates. (DOCX) [file pone.0151969.s001.docx]

**S1 File**

***In vivo* and *in vitro* detection of luminescent and fluorescent *Lactobacillus reuteri* and application of red fluorescent mCherry for assessing plasmid persistence**

Shokoufeh Karimi^1*^, David Ahl^2^, Evelina Vågesjö^2^, Lena Holm^2^, Mia Phillipson^2^, Hans Jonsson^1^, Stefan Roos ^1^

^1^Department of Microbiology, Uppsala BioCenter, Swedish University of Agricultural Sciences, Uppsala, Sweden

^2^Department of Medical Cell Biology, Biomedical Center, Uppsala University, Uppsala, Sweden

Running Head: Luminescent and fluorescent *Lactobacillus reuteri*

*Corresponding author

E-mail: shokoufeh.karimi@slu.se

*Bam*HI

---↓------|---------|---------|---------|---------|---------|

1 *GGATCC*AGATCTAGCGCTATAGTTGTTGACAGAATGGACATACTATGATATATTGTTGCT 60

P11

RBS

---------|---------|---------|---------|---------|---------|

61 ATAGCGTACTTAGCTGGCCAGCATATATGTATTCTATAAAATACTATTACAAGGAGATTT 120

*Nco*I

-----↓----|---------|---------|---------|---------|---------|

121 TAG**CCatgg**ttaagcgggaaaagaatgttatttacggaccagaaccacttcacccacttg 180

1 M V K R E K N V I Y G P E P L H P L E 19

**CBRluc**→

---------|---------|---------|---------|---------|---------|

181 aagatttaactgcaggagaaatgttatttcgggcattacggaaacatagtcaccttccac 240

20 D L T A G E M L F R A L R K H S H L P Q 39

---------|---------|---------|---------|---------|---------|

241 aagcattagttgatgttgtaggagatgaatcattatcttacaaggaatttttcgaagcta 300

40 A L V D V V G D E S L S Y K E F F E A T 59

---------|---------|---------|---------|---------|---------|

301 ctgttcttcttgcacaatcattacataattgtggttacaagatgaatgatgttgtatcta 360

60 V L L A Q S L H N C G Y K M N D V V S I 79

---------|---------|---------|---------|---------|---------|

361 tttgtgctgaaaataatactcgtttctttattcctgttattgctgcatggtatattggaa 420

80 C A E N N T R F F I P V I A A W Y I G M 99

---------|---------|---------|---------|---------|---------|

421 tgattgtagcaccagttaatgaaagttacattcctgatgaattatgcaaagtaatgggta 480

100 I V A P V N E S Y I P D E L C K V M G I 119

---------|---------|---------|---------|---------|---------|

481 tttcaaagccacaaattgtttttactacaaagaatattcttaataaggtattagaagttc 540

120 S K P Q I V F T T K N I L N K V L E V Q 139

---------|---------|---------|---------|---------|---------|

541 aatcacggactaattttattaagcgaattattattcttgatacagttgaaaatattcatg 600

140 S R T N F I K R I I I L D T V E N I H G 159

---------|---------|---------|---------|---------|---------|

601 gatgtgaatctttaccaaattttattagtcgttactcagatggtaatattgctaatttta 660

160 C E S L P N F I S R Y S D G N I A N F K 179

---------|---------|---------|---------|---------|---------|

661 agccacttcactttgatcctgtagaacaagttgctgcaattctttgtagttcaggtacta 720

180 P L H F D P V E Q V A A I L C S S G T T 199

---------|---------|---------|---------|---------|---------|

721 caggattaccaaagggtgttatgcaaactcatcaaaatatttgtgttcgacttattcacg 780

200 G L P K G V M Q T H Q N I C V R L I H A 219

---------|---------|---------|---------|---------|---------|

781 ctttagatccacgttatggtactcaacttattcctggagttacagtacttgtttaccttc 840

220 L D P R Y G T Q L I P G V T V L V Y L P 239

---------|---------|---------|---------|---------|---------|

841 catttttccatgcatttggttttcacattacacttggttactttatggtaggattacgag 900

240 F F H A F G F H I T L G Y F M V G L R V 259

---------|---------|---------|---------|---------|---------|

901 ttattatgtttcgtcggtttgatcaagaagcatttttaaaggcaattcaagattacgaag 960

260 I M F R R F D Q E A F L K A I Q D Y E V 279

---------|---------|---------|---------|---------|---------|

961 tacgttctgttattaatgtacctagtgttattttatttcttagtaagtcaccacttgttg 1020

280 R S V I N V P S V I L F L S K S P L V D 299

---------|---------|---------|---------|---------|---------|

1021 ataagtacgatttatctagtcttcgtgaattatgttgtggtgctgcacctttagctaaag 1080

300 K Y D L S S L R E L C C G A A P L A K E 319

---------|---------|---------|---------|---------|---------|

1081 aagtagcagaagttgctgcaaagcgtttaaatcttccaggaattcggtgtggttttggac 1140

320 V A E V A A K R L N L P G I R C G F G L 339

---------|---------|---------|---------|---------|---------|

1141 ttactgaatctacaagtgctattattcaaactcttggtgatgaatttaagtcaggttctc 1200

340 T E S T S A I I Q T L G D E F K S G S L 359

---------|---------|---------|---------|---------|---------|

1201 ttggacgggttactccattaatggctgcaaaaattgctgatcgagaaacaggaaaggcac 1260

360 G R V T P L M A A K I A D R E T G K A L 379

---------|---------|---------|---------|---------|---------|

1261 ttggacctaatcaagttggtgaattatgtattaagggaccaatggtttcaaagggttatg 1320

380 G P N Q V G E L C I K G P M V S K G Y V 399

---------|---------|---------|---------|---------|---------|

1321 taaataatgttgaagctactaaggaagcaattgatgatgatggatggttacatagtggtg 1380

400 N N V E A T K E A I D D D G W L H S G D 419

---------|---------|---------|---------|---------|---------|

1381 attttggatactacgatgaagatgaacacttttatgttgtagatcggtacaaggaactta 1440

420 F G Y Y D E D E H F Y V V D R Y K E L I 439

---------|---------|---------|---------|---------|---------|

1441 ttaagtacaagggtagtcaagttgctcctgcagaattagaagaaattttacttaagaatc 1500

440 K Y K G S Q V A P A E L E E I L L K N P 459

---------|---------|---------|---------|---------|---------|

1501 catgtattcgagatgttgcagttgtaggtattccagatttagaagctggtgaattacctt 1560

460 C I R D V A V V G I P D L E A G E L P S 479

---------|---------|---------|---------|---------|---------|

1561 cagcatttgttgtaaaacaaccaggtactgaaattacagctaaggaagtatacgattacc 1620

480 A F V V K Q P G T E I T A K E V Y D Y L 499

---------|---------|---------|---------|---------|---------|

1621 ttgcagaacgtgtttctcatactaagtatttacgaggtggagtacgttttgttgatagta 1680

500 A E R V S H T K Y L R G G V R F V D S I 519

---------|---------|---------|---------|---------|---------|

1681 ttccacggaatgttactggaaagattacacgaaaagaattattaaaacaattattagtaa 1740

520 P R N V T G K I T R K E L L K Q L L V K 539

*Mlu*I RBS *Sna*BI

--------|--------↓-|---------|---↓------|---------|---------|

1741 aagcaggtggataa*ACGCGT*AAGGAGATTT***TACGTa***tggtttctaagggtgaagaagata 1800

540 A G G * M V S K G E E D N 16

**mCherry→**

---------|---------|---------|---------|---------|---------|

1801 atatggctattattaaggaatttatgcgttttaaggtacatatggaaggtagtgttaatg 1860

17 M A I I K E F M R F K V H M E G S V N G 36

---------|---------|---------|---------|---------|---------|

1861 gtcatgaatttgaaattgaaggtgaaggagaaggtcggccatatgaaggtactcaaactg 1920

37 H E F E I E G E G E G R P Y E G T Q T A 56

---------|---------|---------|---------|---------|---------|

1921 ctaagttaaaggttactaaaggtggaccattaccttttgcatgggatattctttcaccac 1980

57 K L K V T K G G P L P F A W D I L S P Q 76

---------|---------|---------|---------|---------|---------|

1981 aatttatgtacggatctaaggcttatgttaagcacccagcagatattcctgattacctta 2040

77 F M Y G S K A Y V K H P A D I P D Y L K 96

---------|---------|---------|---------|---------|---------|

2041 aacttagttttcctgaaggttttaagtgggaacgagttatgaattttgaagatggtggag 2100

97 L S F P E G F K W E R V M N F E D G G V 116

---------|---------|---------|---------|---------|---------|

2101 ttgtaactgtaacacaagatagttcattacaagatggagaatttatttacaaggttaagt 2160

117 V T V T Q D S S L Q D G E F I Y K V K L 136

---------|---------|---------|---------|---------|---------|

2161 tacgtggaactaattttccatcagatggtcctgtaatgcaaaagaaaactatgggttggg 2220

137 R G T N F P S D G P V M Q K K T M G W E 156

---------|---------|---------|---------|---------|---------|

2221 aagctagtagtgaacgtatgtacccagaagatggagcattaaagggtgaaattaagcaac 2280

157 A S S E R M Y P E D G A L K G E I K Q R 176

---------|---------|---------|---------|---------|---------|

2281 ggcttaaacttaaggatggtggacattacgatgctgaagttaagactacatacaaggcaa 2340

177 L K L K D G G H Y D A E V K T T Y K A K 196

---------|---------|---------|---------|---------|---------|

2341 aaaagccagttcaattacctggtgcttacaatgtaaatattaagttagatattacttctc 2400

197 K P V Q L P G A Y N V N I K L D I T S H 216

---------|---------|---------|---------|---------|---------|

2401 acaatgaagattacacaattgttgaacaatacgaacgggcagaaggtcggcactctactg 2460

217 N E D Y T I V E Q Y E R A E G R H S T G 236

*Xho*I

---------|---------|---------↓|--

2461 gaggaatggatgaactttacaaataa***CTCGAG*** 2492

237 G M D E L Y K * 243

**FIG A. Nucleotide sequence and corresponding amino acid sequence of the CBRluc-mCherry casette.**

The whole cassette, including the constitutive promoter (P11), is 2492 bp. The P11 fragment flanked by *Bam*HI and *Nco*I is 123 bp, the CBRluc fragment flanked by *Nco*I and *Mlu*I is 1631 bp and the mCherry fragment flanked by *Sna*BI and *Xho*I is 714 bp. Features of the sequence are specified as follows: nucleotide sequence with underlined capital letters = P11 promoter region; bold underlined nucleotide sequence with capital letters = ribosomal binding sites; italic letters = restriction enzyme cleavage sites.


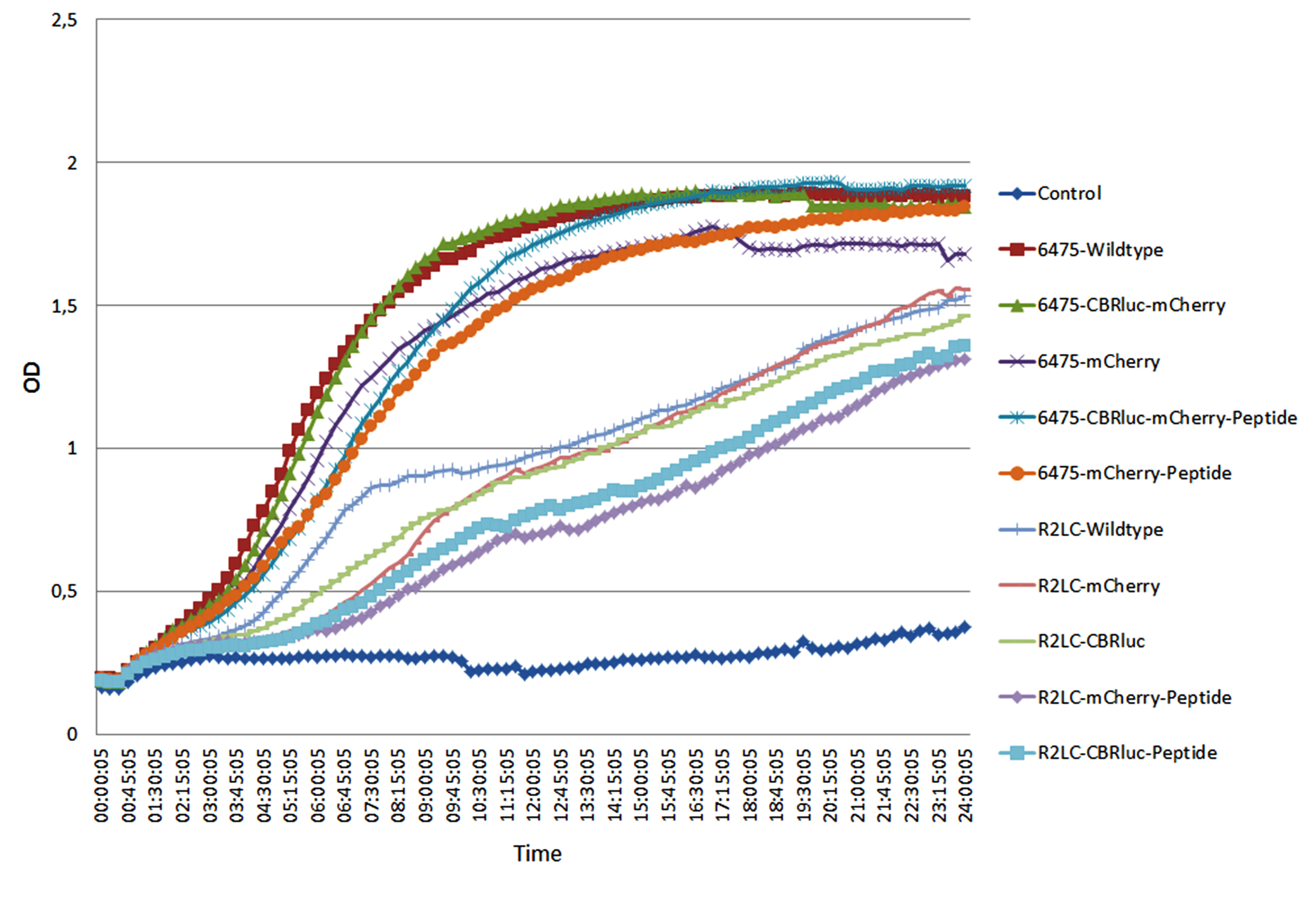


**FIG B. Growth curves of recombinant and wildtype strains.**

The growth of 6475-CBRluc-mCherry, 6475-mCherry, R2LC-mCherry and R2LC-CBRluc was compared with that of wildtype strains ATCC PTA 6475 and R2LC in the presence or absence of SppIP inducing peptide.


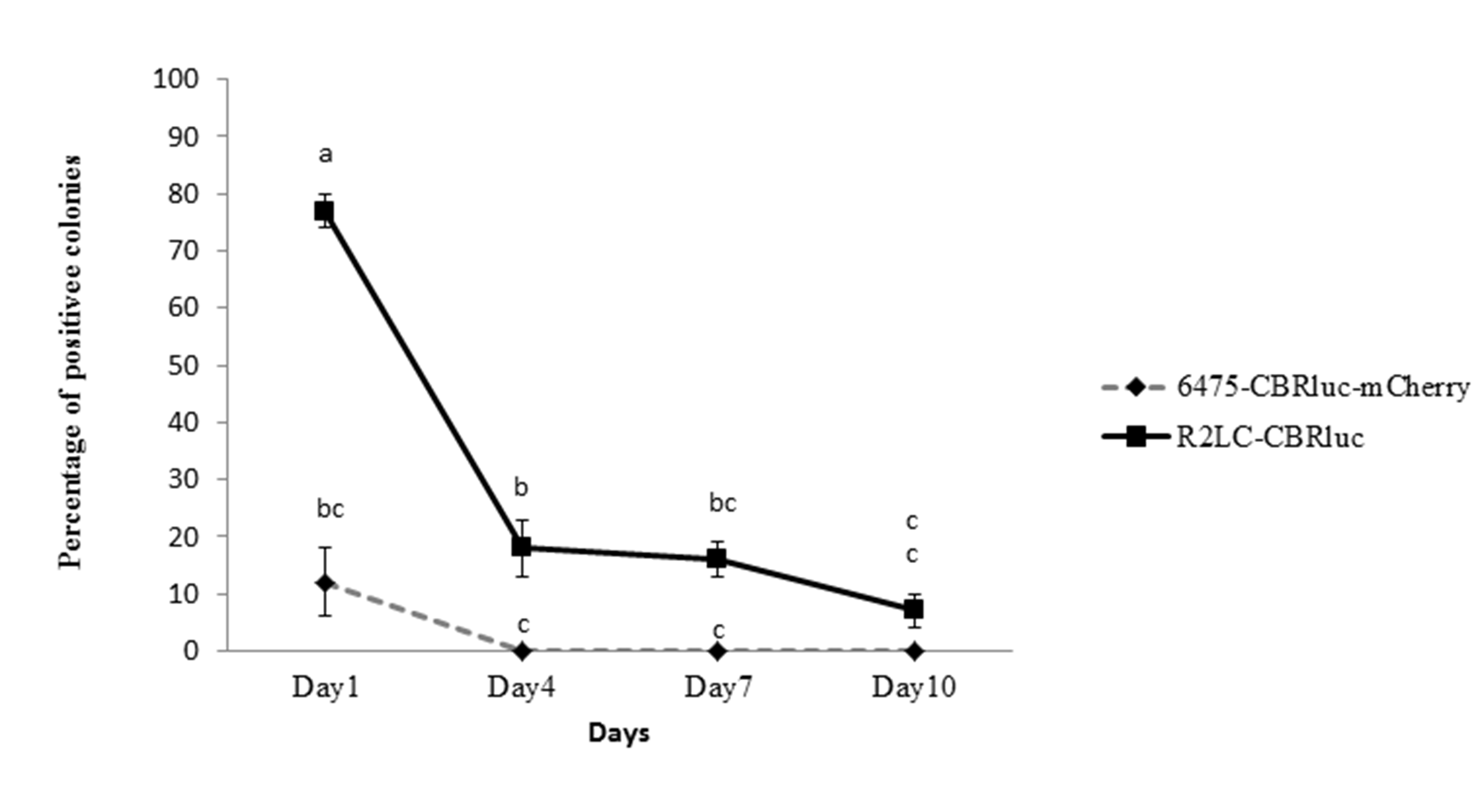


**FIG C. Measurment of plasmid stability using replica plating.**

6475-CBRluc-mCherry and R2LC-CBRluc were cultured serially in MRS broth without selection pressure for 10 days. Samples from days 1, 4, 7 and 10 were replica-plated to evaluate the plasmid persistence.

**
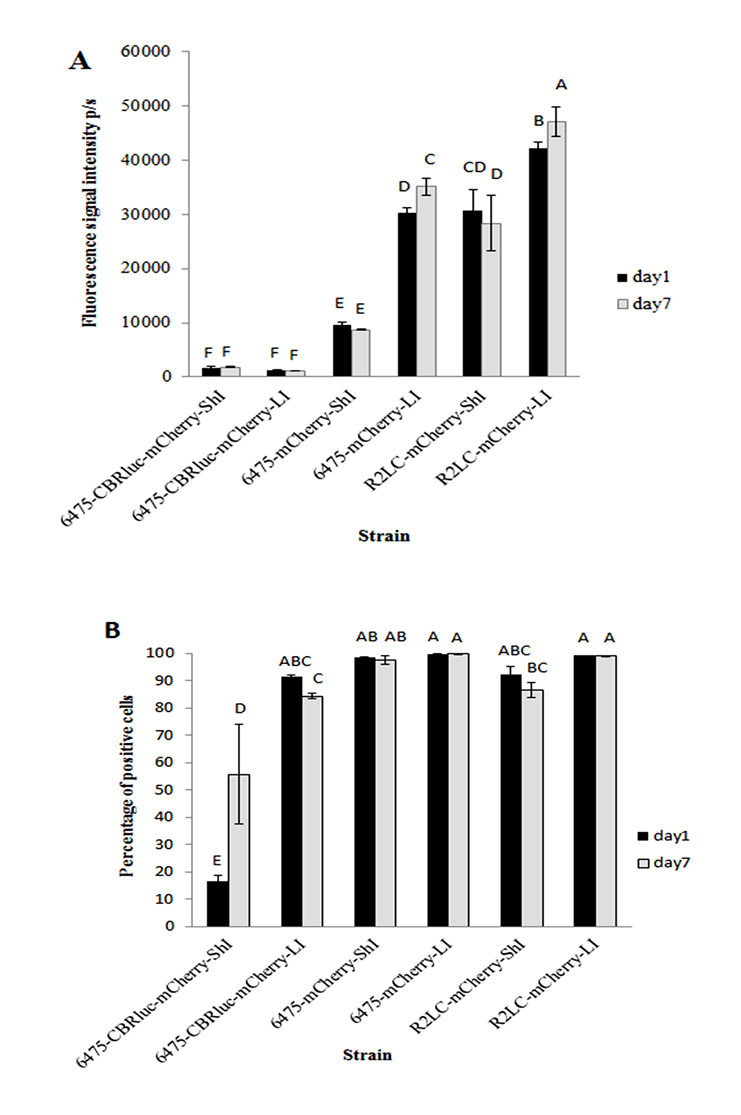
**

**FIG** D. **Combined effect of induction period and subculture on mCherry-producing strains.** (**A)** Fluorescence signal intensity. (**B)** Percentage of mCherry-producing bacteria. Samples from days 1 and 7 of a serial subculture in the presence of antibiotics were analyzed by flow cytometry after a short or long induction period. Columns labelled with different letters are significantly different (*p*≤0.05). The error bars indicate the standard deviation of median values obtained from five independent biological replicates.
